# Bioactivity Screening of Mulberry Leaf Extracts and two Related Flavonoids in Combination with Cisplatin on Human Gastric Adenocarcinoma Cells

**DOI:** 10.22037/ijpr.2020.1101087

**Published:** 2020

**Authors:** Ghazaleh Ghavami, Samad Muhammadnejad, Saeid Amanpour, Soroush Sardari

**Affiliations:** a *Drug Design and Bioinformatics Unit, Medical Biotechnology Department, Biotechnology Research Center, Pasteur Institute of Iran, Tehran, Iran. *; b *Cell-Based Therapies Research Center, Digestive Disease Research Institute, Tehran University of Medical Sciences, Tehran, Iran. *; c *Cancer Biology Research center, Tehran University of Medical Sciences (TUMS), Tehran, Iran*

**Keywords:** Mulberry, Cisplatin, Rutin, Isoquercetin, Combination effect, Gastric cancer

## Abstract

The successful therapy strategy of gastric cancer is defined as devastating the cancerous cells without exposing systematic toxicity and undesirable side effects. One strategy to overcome cancer treatment related difficulties could be combination therapy with natural products with anticancer drugs to introduce effective antitumor effects in addition to reduce undesirable side effects. In this regard, different extracts of mulberry leaf, isoquercetin and rutin as the extracted flavonoids from *Morus alba*, mulberry, in single dose as well as in combination with cisplatin against gastric cancer cell line were applied. This innovative treatment led to cytotoxic effect on gastric cancer cells in a synergistic manner. The findings anticipated that these herbal products have exceptional potential for future gastric cancer investigations and therapy.

## Introduction

Cancer with approximately 14 million new cases and 8 million deaths in 2012 has been known as a major cause of morbidity and mortality around the world. The relevant predictions demonstrated that 22 million new cancer cases and 13 million cancer related deaths would happen by 2030 annually (1). Additionally, gastric cancer has been reported as the third leading cause of death among various cancer types globally in 2016 (2). 

The aim of successful treatment of gastric cancer such as other cancers is defined as eliminating the tumor cells without harming the normal cells. The most general types of cancer treatment strategies include surgery, radiation, and chemotherapy which can be utilized either alone or in combination with each other or other therapies.

Chemotherapy as one of the major cancer treatment strategies is the function of chemical/natural based compounds for killing tumor cells, and its effects are systemic with exposing a wide range of side effects (3).

Based upon the current guidelines, first line chemotherapeutic therapy involves a platinum agent based double, *e.g.* cisplatin or carboplatin in combination with a third-generation cytotoxic drug (3). 

Cisplatin has been used as the standard treatment strategy for a wide range of cancers such as gastric cancer (4). Unfortunately, the benefit of cisplatin is reduced because of exposing a wide range of side effects such as neurotoxicity in addition to damaging cardiovascular and nervous systems. One of the strategies in novel cancer research is presenting creative treatments having the same therapeutic properties of the reference drug, but with less systemic side effects.

Compounds from natural resources and mainly herbal compounds have a long history as the source of anticancer drugs (3). Herbal medicines are commonly low in cost, bountiful, and demonstrate little side effects in clinical practice. The tumor preventive and protective effects of the herbal compounds are related to their properties on cellular defense such as detoxifying and antioxidant phenomena in addition to the stimulation of anti-inflammatory and antitumor or anti-metastasis reactions (3).

In clinical therapy procedures, most patients respond defectively to conventional chemotherapy due to undesirable anticancer drug resistance process. Consequently, it is critical to introduce novel treatment strategies to progress the sensitivity of tumor cells to chemotherapy induced cell death in the frame of combination chemotherapy to target tumors. This is usually done with different possible mechanism towards enhancing the rate of chemotherapy efficacy parallel to reducing the relevant undesirable side effects (4). 

The previous research findings have demonstrated that natural medicine can play critical role in combination cancer chemotherapy approaches. Mulberry (*Morus alba* L.) is one of the natural medicine sources which is generally distributed in Asia and its leaves have been utilized as a medicine since ancient times.

The leaf and leaf-derived extracts of *M. alba* have been utilized as food or medicine in many countries. The leaves are utilized in decreasing the risk and treatment of type 2 diabetes in addition to cardiovascular, urinary, and nervous related diseases (5-6). Recently, a wide range of investigations has been specified to cancer prevention and treatment properties of *M. alba* (5-6).

In the current research, the antioxidant properties and anticancer effects of ethyl acetate, chloroformic and ethyl acetate fractions of *M. alba* leaf alone and in combination with cisplatin against AGS (human Caucasian gastric adenocarcinoma) cell line were evaluated *in-vitro*. Furthermore, the combinational anticancer effects of cisplatin with two natural compounds isoquercetin and rutin were investigated.

## Experimental

*Preparation of herbal extracts and compounds*

*M. alba *leaves were gathered form Neyshabur, Razavi Khorasan Province, Iran (36°12’11.5”N 58°48’02.8”E). Voucher herbarium sample of the plant was prepared and is accessible. The leaves were dried at room temperature and mechanically powdered. The dried plant powder was extracted by ethanol (Hamonteb, Iran) (80%) using percolation method (7-8). The extracts were then gathered, pooled, and dried by rotary evaporator (IKA™ RV05 Basic, USA). To provide water, ethyl acetate and chloroformic fractions of *M. alba *leaf, 5 gr of dried crude extract (for each extraction procedure) was extracted by liquid–liquid extraction manner using water-ethyl acetate (BioChem, France) and water-chloroform (BioChem, France). The extracts were evaporated and after drying, 10 mg of each sample (crude, water, ethyl acetate and chloroform extracts) was dissolved at DMSO (Fluka, USA) plus RPMI 1640 (Biosera, France) as stock, after that extract dilution was performed with RPMI 1640 (Biosera, France). Solutions of isoquercetin (Sigma-Aldrich, Germany) and rutin (Sigma-Aldrich, Germany) were provided as mentioned above. Cisplatin (Mylan, Netherlands) was diluted with RPMI 1640 as the anticancer drug.

*Cell culture*

AGS as known Human Gastric Adenocarcinoma cell line (IBRC C10071) was provided from *Iranian Biological Resource Center* (Tehran, Iran). They were plated in 25 and 75 cm^2^ cell culture flasks in RPMI 1640 plus 10% Fetal Bovine Serum (Biosera, France) and 2 g/l HEPES buffer (Biosera, France), under a 5% CO_2_ atmosphere with pH 7.4. The cells with 70-80% of confluence were removed by trypsin-EDTA solution 1X (Biosera, France). For each test, all of the cells with density of 10^4^ cells/cm^2^ in 96-multiwell plates were seeded for MTT and 10^5^ cells/cm^2^ in 24-multiwell plates for cell cycle and cell proliferation tests (three wells for each sample and treatment), in that order. 

*MTT cell assay*

The toxicity properties of the herbal extracts and the related compounds on the gastric cancer cells were assessed by of MTT (3-(4,5-dimethylthiazol-2-yl)-2,5-diphenyltetrazolium bromide; thiazolyl blue) test (Sigma-Aldrich, Germany). The cells with RPMI 1640 were adjusted to 1.0×10^4^ cells/cm^2^ to plate in 96-well plates (200 μL/well) for incubation time 24 h under a 5% CO_2_ atmosphere. Following treatment with crude, water, ethyl acetate, and chloroformic extracts (40-5000 µg/mL) as well as cisplatin (0.8-500 µg/mL), isoquercetin and rutin (8-1000 µg/mL) for 48 h incubation time, each well was obtained 20 μL of MTT (5 g/l) and incubated during 4 h. After removing the medium and adding 100 μL/well DMSO, the plates were analyzed by multiwell scanning spectrophotometer (ELISA reader, Organon Tekninka, Netherlands) in 545 nm (9). The percentage of the cell toxicity was obtained by the following equations: %Toxicity = (1 − average absorbance value of experimental group/average absorbance value of control group) ×100% 

In addition, based on MTT assay, the effects of cisplatin (2 and 10 µg/mL), crude (100 and 200 µg/ml), ethyl acetate (80 and 160 µg/mL), and chloroformic extracts (160 and 320 µg/mL) of *M. alba *leaf as well as isoquercetin (40 and 80 µg/mL) and rutin (40 and 80 µg/mL) in single doses and combination manner on AGS cell line were evaluated, as the mentioned.

*Analysis of cell cycle*

Isoquercetin, rutin, crude, ethyl acetate, and chloroformic extracts in the single doses and in combination with cisplatin in their IC_50_s doses were used to treat the gastric cancer cells (1.0×10^5^ cells/cm^2^) during 48 h. After that, the treated AGS cells were washed in cold PBS (Biosera, France), fixed in cold 70% ethanol (Sigma-Aldrich, Germany) and kept at −20 °C for 6 h. Before analysis, the cells were washed twice in PBS, resuspended in staining solution (final concentration 0.1% Triton X-100, 0.5 mg/Libonuclease A (Sinaclon, Iran) and 0.025 mg/ml propidium iodide (Sigma-Aldrich, Germany) to incubate in the dark at room temperature for 30 min and analyze by CyFlow (Partec, Germany) (10). 

*Annexin V/PI double staining assays*

The cells incubated under various treatments for 48 h (1.0×10^5^ cells/cm^2^) with isoquercetin, rutin, crude, ethyl acetate and chloroformic extracts single and in combination with cisplatin in their IC_50_ doses (24-well plates), the non-adherent cells were collected, and the adherent cells were quickly washed with PBS buffer and were taken away with trypsin-EDTA solution 1X at room temperature. Subsequently, the procedure was performed based on the manufacturer staining instruction for Fluorochrome annexin V (FITC) and Propidium iodide (PI) solutions (IQ Products BV, Netherlands). Then, the results were analyzed by a flow cytometer as CyFlow (Partec, Germany) (11).

*Antioxidant property measurement by DPPH method*

DPPH (1,1-diphenyl-2-picrylhydrazyl) method was carried out to evaluate the antioxidant properties of the mulberry crude, ethyl acetate, and chloroformic extracts. The stock solution of DPPH was prepared at a concentration of 200 μM in absolute methanol (Sigma-Aldrich, Germany) with final concentration of 10 mg/mL for each herbal samples. DPPH was added into 96-well plate (100 μL/well) and at this, each well received 100 μL of the plant extracts (concentrations of 7.8-1000 μg/mL). Methanol alone and with DPPH was used as blank and negative control and ascorbic acid as positive control, in that order. After 30 min incubation the treated wells (three wells for each sample and treatment) in the dark, the relevant absorbance was measured at 517 nm by scanning spectrophotometer (ELISA reader, Organon Tekninka, Netherlands).

Finally, the antioxidant activity percentage (*AA*%) was analyzed from the ratio of decreasing absorbance of the sample solution (*A*_0_ – *A*_s_) to absorbance of the blank DPPH solution (*A*_0_). The *AA*% was utilized to calculate the median inhibitory concentration (IC_50_) via dose response curves (12).

*HPLC analysis of the bioactive fraction*

For further investigations of the most bioactive fraction as ethyl acetate fraction of *M. alba* leaf extract to evaluate its photochemical fingerprint towards further biopharmaceutical studies, the mentioned plant extract was examined by HPLC (Knauer, Germany). The ethyl acetate extract (20 µL injection volume) was evaluated based on using the mobile phase (BioChem, France) involving combination of acetonitrile and water (pH = 2.5 with orthophosphoric acid) in gradient manner (90% acetonitrile -100% water - 25% acetonitrile in 60 min) by Nucleodur C18 column (250 cm*4mm), flow rate 0.8 in 290 nm (room temperature). 

*Statistical analysis*

The presented data were mean ± Standard error of mean (SEM)/ ± Standard deviation (SD) of three independent experiments performed in duplicate (n = 6) and IC_50_ was examined by Graph Pad Prism 5.0 program (GraphPad, La Jolla, CA, USA) with the 95% confidence interval. Statistical comparisons were carried out by ANOVA and *post hoc* Tukey’s test. The differences were considered significant when *p *< 0.05.

To analyze the interaction between herbal extracts and the anticancer drug, CI method was used. CI was calculated by ComboSyn (ComboSyn Inc., NY, USA) based on the combination index theorem of Chou and Talalay. This method presents a quantitative measure based on the mass-action law of the degree of drug interaction in terms of Synergism (CI<1), Additive (CI*=*1) and Antagonism (CI*>*1) for a particular endpoint of the effect measurement. CI was resulted from n(CI)x=Σn j=1(D)j/(D50)j in which D and D_50_ are defined as Dose and Median-effect dose and the dose produces 50% effect as IC_50 _. 

## Results

*MTT cell assay*

The MTT assay was utilized for measurement of cell toxicity caused by plant samples and cisplatin in single (Table 1) and combination manners (Table 2).

*Analysis of cell cycle*

To examine the effects of single and combination doses of herbal samples (IC50 dose) in addition to cisplatin on gastric cancer cell line* (*Table 3)*,* the DNA-binding dye include propidium iodide was used to analysis occurring changes in cell cycle phases by quantitation of DNA content.

*Annexin V/PI double staining assays*

**Figure 1:**
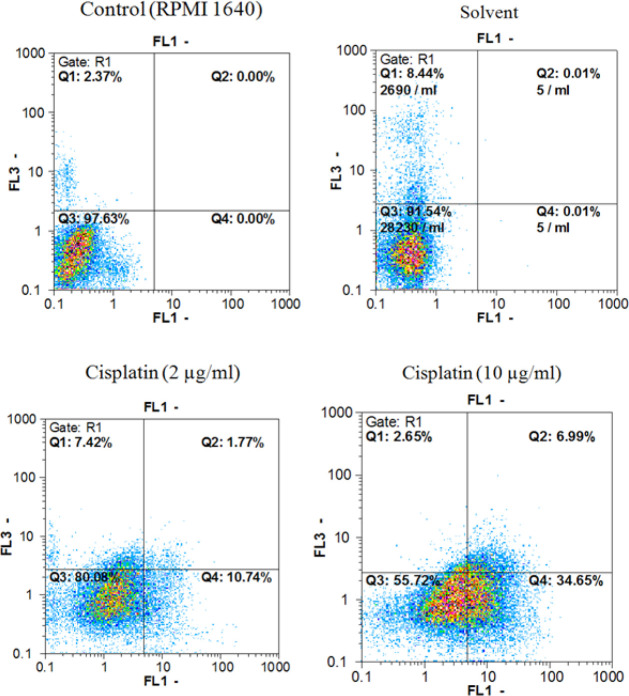
Findings in related to Annexin V-FITC *vs*. propidium iodide quantitation of AGS cells treated with the solvent, cisplatin (2 and 10 µg/mL) doses in addition to non treated cells (RPMI 1640).

**Figure 2.**
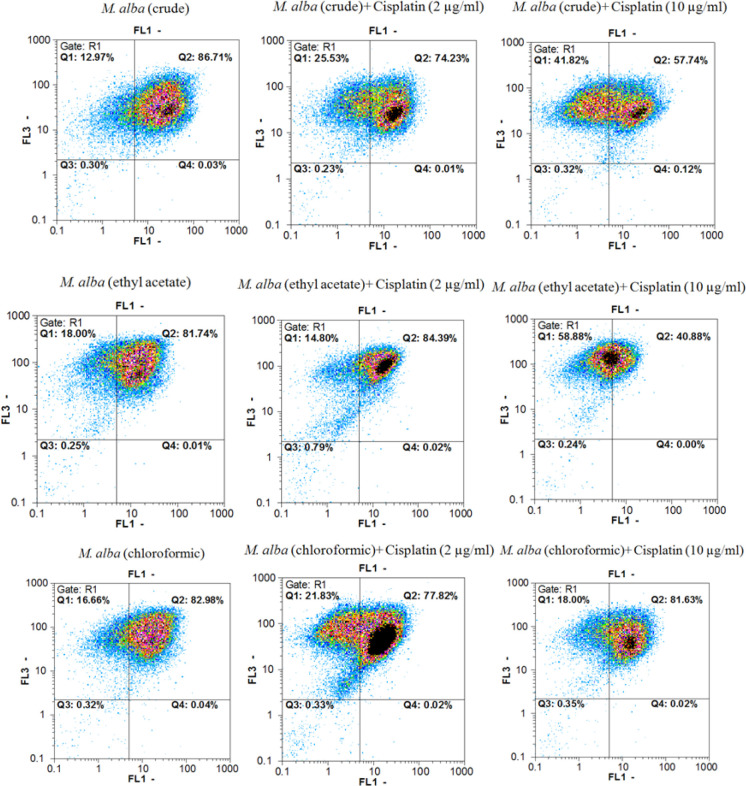
Findings in related to Annexin V-FITC *vs*. propidium iodide quantitation of AGS cells treated with *M. alba* extracts in addition to cisplatin (single and combination manner). FL1 and FL2 channels are related to Annexin V and FITC

**Figure 3:**
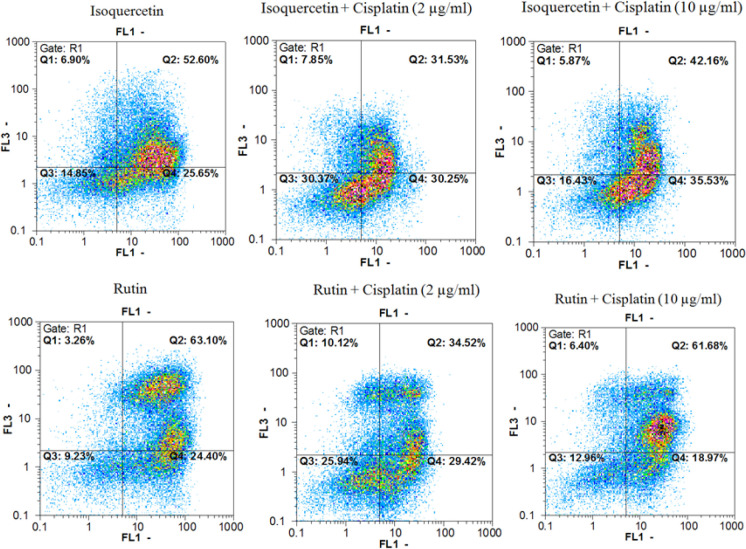
Findings in related to Annexin V-FITC *vs*. propidium iodide quantitation of AGS cells treated with natural compounds (isoquercetin and rutin) to cisplatin (single and combination manner). FL1 and FL2 channels are related to Annexin V and FITC

Annexin V-FITC *vs*. propidium iodide quantitation method was used to determine the properties of herbal extracts/compounds in single (two times the IC_50_) and combination (IC_50_) doses with cisplatin (2 and 10 µg/mL) towards inducing apoptosis or/and necrosis (Figure 1-3) on AGS cell line.

*Antioxidant property measurement by DPPH method*

Free radical scavenging activities of *M. alba* extracts were determined via DPPH method.

**Figure 4:**
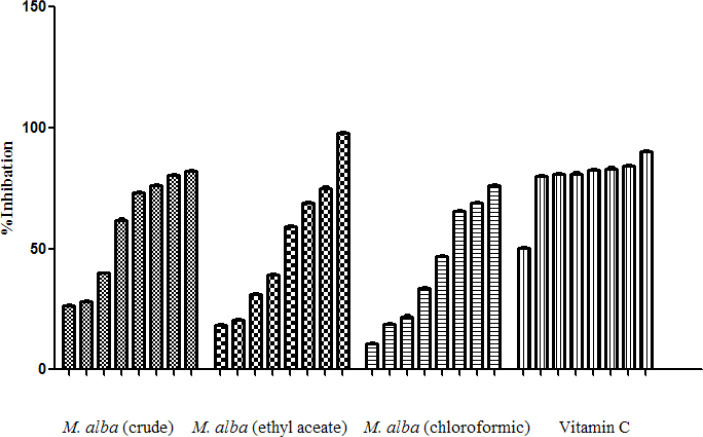
Free radical scavenging abilities of *M. alba* extracts based on DPPH method, each column in graph is referred to the relevant concentration

In fact, the free radical scavenging activities of the extracts in related to their ability of antioxidant compounds to lose hydrogen were examined (Table 4), (Figure 4).

*HPLC analysis of the bioactive fraction*

**Figure 5:**
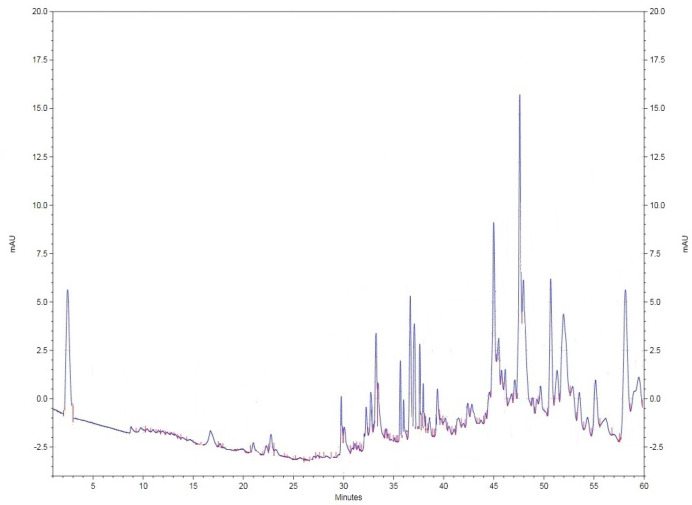
HPLC based phytochemical fingerprinting of ethyl acetate fraction of *M. alba* extract

The ethyl acetate fraction of *M. alba* as the most bioactive extract known during current research was selected for evaluation via HPLC method towards identifying its photochemical fingerprint (Figure 5).

## Discussion

Chemotherapy is a broadly accepted form of gastric cancer therapy and there is growing interest in its application in earlier stages of the cancer particularly when combined with other therapy approaches. Platinum drug based chemotherapy is one of the cancer therapy strategies which has been widely used in the patients with gastric cancer (3).

Actually, platinum drugs are still of crucial importance based on clinical investigations and the outcomes of meta-analyses, despite their undesirable side effects such as exposing systematic toxicity and the inherent resistance. Therefore, of overcoming the pointed challenges of efficient gastric cancer chemotherapy, a wide range of relevant investigation have been focused on the combinational therapy strategies. 

The recent research demonstrated that utilizing natural compounds along with chemotherapy drugs in the patients with advanced solid tumors could decrease the side effects caused by chemotherapy, and this could be a critical strategy to cancer control and treatment (3).

Moraceae family as one of the medicinal plants has been known traditionally because of their resourceful applications in different aspects such as agriculture, cosmetic and food in addition to pharmaceutical industries. Their biomedical significance is related to their wide range of pharmacological properties due to treatment of diverse inflammatory, cancer, metabolic, infectious, and gastrointestinal diseases (5-6).

Bioactivity guided fractionation of Moraceae herbal family has made the discovery of diverse bioactive phytochemical and secondary metabolites involving in this family. Findings from previous investigations have verified that the aqueous, methanolic, and ethanolic extracts of the belonging species and their bioactive compounds demonstrate significant antioxidative, antidiabetic, antistress, nephroprotective, antimicrobial, antimutagenic, anticancer, anxiolytic, hepatoprotective, anthelmintic, antimicrobial, immunemodulatory, and cholesterol lowering properties (5-6).

*M. alba *is the white mulberry from Moraceae and also famous as Tout in Iran and India. This known herb with broad distribution has been traditionally utilized for treatment of different diseases such as asthma, cough, bronchitis, edema, insomnia, wound healing, diabetes, influenza, eye infections, and nosebleeds (13). 

*M. alba *is a rich source of ascorbic acid, carotene, vitamin B1, folic acid, folinic acid, isoquercetin, quercetin, tannins, flavonoids and saponins (13). Additionally, white mulberry leaf contains triterpenes (lupeol), sterols (β-sitosterol), bioflavonoid (rutin, moracetin, quercetin-3-triglucoside and isoquercitrin), coumarins, volatile oil, alkaloids, 1-deoxynojirimycin, amino acids, and organic acids.

Previous phytochemical screening of *M. alba* fruit and leaf extract has revealed significant antiproliferative efficacy against various cancer cells lines involving Calu-6, MCF-7, HCT-116 and SNU-601 (6).

Two flavonoids, quercetin-3-*O*-*β*-D-glucopyranoside, and quercetin-3-7-di-*O*-*β*-D-glucopyranoside were isolated from the aqueous methanol leaf extract of *M. alba* inhibiting the growth of human leukemia HL-60 cells (14)*. *Also, a flavanone glycoside isolated from the root bark of *M. alba *displays antiproliferative activity against HO-8910 cell line (14)*. *Additionally, albanol A from the root bark of *M. alba* showed cytotoxic and apoptotic activities in human leukemia HL-60 cells with inducing early apoptosis based on significant diminution in procaspases-3, -8, and -9, and activation of caspase-2 (14)*. *Also*, *isolated morusin from the root bark of mulberry stimulates apoptosis and suppresses NF-*κ*B in HT-29 cells (14).

Another investigation demonstrated the anticancer property of the methanol root bark extract of mulberry with simulating cell growth arrest and apoptosis in SW480 cells based upon degradation of ROS-dependent cyclin D1 proteasomal and expression of ROS/GSK3*β*-dependent ATF3 (14). Furthermore, the isolated 11 flavonoids from the methanol leaf extract of mulberry demonstrated cytotoxic property against HeLa, MCF-7, and Hep-3B cells (14). 

Moreover, methanol leaf extracts of *M. alba *displayed considerable inhibitory property on the proliferation of HepG2 cells with suppressing nuclear factor kappa B gene expression and major refuses in *α*-fetoprotein, *γ*-glutamyl transpeptidase, and alkaline phosphatase in HepG2 (14-15)*. *

In another study, the synergistic effect of flavonoid extract of white *M. alba* leaf in combination with doxorubicin was proved with significant reducing in Bcl2 level and an enhancing in Bax level in A-172 GBM cell line (16).

Indeed, the previous investigations finalized by research group that has been involved in current study, demonstrated the inhibitory effects of mulberry leaf crude extract against six cancerous cell lines as AGS, MCF7, SW742, SKLC6, A375, and PLC/PRF/5 with significant less effective toxicity dose than HFFF normal cells. In addition, the synergistic property of the *M. alba* leaf crude extract with cisplatin and doxorubicin against drug resistance tumor cells-ACHN and A2780/cp cell lines, were evaluated by the pointed research group (17-18). On the other hand, the reported findings showed the significant anticancer effects of isoquercetin and rutin as the major flavonoid isolated from *M. alba* (19)*. *

As another significant point, it would be notable that during the previous study, *in-vivo* investigated the acute toxicity of *M. alba* leaf ethanol extract when intraperitoneally administered. The relevant findings cleared that the mulberry leaf ethanol extract was not genotoxic with highly inhibitory property against acute inflammation that confirmed safety of the medicinal use of *M. alba* leaves (20).

Three of the identified flavones are quercetin, an aglycone structure broadly found in nature, rutin as a hydrophilic molecule in addition to isoquercetin, a naturally stirring glycoside of quercetin. The last *in-vitro* and *in-vivo* investigations evaluated the antioxidants, antiinflammatory, antiallergic, and antiproliferative effects of isoquercetin against fibrosarcoma, prostatic, pancreatic, breast, cerebral, lymphoproliferative, colon, and liver cancers (21). In addition, the relevant findings demonstrated the synergistic property of isoquercetin with anticancer drug sunitinib against renal cell carcinoma. In fact, isoquercetin was extremely safe, with a significant activity in terms of improvement of sunitinib adverse concept in phase I trial (22).

A wide range of pharmacological properties such as antioxidant, cytoprotective, vasoprotective, anticarcinogenic, neuroprotective, and cardioprotective activities in addition to anticancer effects against leukemia, colon cancer, neuroblastoma, colorectal pulmonary metastasis, melanotic melanoma, and hepatic carcinoma have been reported for rutin as a flavonol glycoside (23). Moreover, previous data cleared that rutin arrested cell cycle at G2/M and G0/G1 phases towards simulating cell apoptosis. Indeed, rutin by nonselective embarrassment of P-gp and BCRP pumps reversed multidrug resistance and renovated chemosensitivity to cyclophosphamide of human chemoresistant in triple-negative breast cancer cells, competently (24).

On the subject of mentioned facts in addition to other investigations demonstrateing the nephroprotective property of leaf extract and flavonoid isolated from *M. alba *on cisplatin-induced nephrotoxicity in the rats (25), the neuroprotection ability of mulberry leaf extract against glyphosate-induced toxicity (26), and the effect of rutin as a neuroprotective agent as well as protection property of rutin on optic nerve against cisplatin-induced toxicity (27-28), the current research was planned. 

Findings from MTT assay demonstrated that ethyl acetate fraction of *M. alba* leaf extract with IC_50_ 62.51 µg/mL was the most bioactive fraction in inducing toxicity on gastric cancer cell line. Moreover, it was cleared that the water fraction was the less activity that led to removing the water extract from further tests in the current research. 

Indeed, analyzing the combination effects of isoquercetin, rutin, crude, ethyl acetate, and chloroformic fractions of *M. alba* leaf extract with cisplatin was cleared that the mentioned plant extracts/compounds induced the toxicity effect of cisplatin in its IC_50 _and less than IC_50 _doses in synergistic manner (CI<1). The relevant results based on calculated CIs showed that the ethyl acetate fraction (80 and 160 µg/mL doses) can simulate anticancer effect of cisplatin in both tested doses in comparison with isoquercetin and rutin as well as other examined extracts in the current study.

Analysis of the cell cycle based on quantitation of DNA contentwas confirmed the properties of isoquercetin, rutin, crude, ethyl acetate, and chloroformic fractions (IC_50 _doses) in reducing cell count percentage in G0/G1, S, and G2/M phases as well as inducing it in sub G0 phase (dead cells) in comparison with non-treated cells (RPMI 1640) and solvent treated cells.

Moreover, the pointed data demonstrated that isoquercetin, rutin, and the entire *M. alba* leaf extracts in combination with cisplatin (IC_50 _and less than IC_50 _doses) could decrees cell count percentage in G0/G1, S, and G2/M phases in addition to increase it in sub G0 phase in comparison with the single doses of cisplatin. 

In addition, data from evaluating the *M. alba* leaf extracts, isoquercetin and rutin in single (two times the IC_50 _dose) and combination (IC_50 _dose) doses with cisplatin (2 and 10 µg/mL) towards inducing pre-apoptosis, apoptosis and necrosis percentages on gastric cancer cells showed that the combination treatment of AGS cells with cisplatin plus the entire tested extracts and natural compounds could raise the pointed percentages in comparison with the single dose treatment of cisplatin.

Besides, free radical scavenging activities of *M. alba* leaf extracts were examined based on DPPH method and cleared that ethyl acetate fraction of *M. alba* leaf extract with IC_50_ 32.578 µg/mL was the most active fraction. The phytochemical fingerprint of the ethyl acetate fraction was evaluated by HPLC method based on the obtained facts as its highest potency among the examined extracts.

Findings during the current research showed the possible anticancer properties of *M. alba* leaf extracts especially the ethyl acetate fraction, as well as isoquercetin and rutin against gastric cancer cells. As the important view, these findings cleared that combination usage of cisplatin with isoquercetin, rutin, and ethyl acetate fraction of *M. alba* leaf extract can introduce innovative gastric cancer therapy strategy with profitable efficacy parallel to the minimum systematic side effects caused by the current chemotherapy regimen using cisplatin in single manner. 

**Table 1:** The toxicity effects of samples on AGS cell line

**Samples **	**IC**_50_ ** (µg/mL)** ^1^	**Statistical comparison with the solvent (** ***p *** **< 0.05)**
*M. alba* (crude)	103.49 ± 0.75	*** *p *< 0.001
*M. alba* (ethyl acetate)	62.51 ± 0.67	*** *p *< 0.001
*M. alba* (chloroformic)	151.33 ± 0.96	*** *p *< 0.001
*M. alba* (Water)	892 ± 0.99	* *p *< 0.05
Isoquercetin	31.8 ± 0.68	*** *p *< 0.001
Rutin	14.87 ± 0.74	*** *p*<0.001
Cisplatin	10.58 ± 0.57	-

^1^ Data shown as mean ± SEM

**Table 2:** The combination effects of herbal samples with cisplatin on AGS cell line

Samples	**Herbal dose (µg/mL)**	**Cisplatin dose (µg/mL)**	**CI**	**Fa**
*M. alba* (crude) plus cisplatin	200.0	10.0	0.313	0.84
	100.0	10.0	0.669	0.39
	200.0	2.0	0.307	0.84
	100.0	2.0	0.700	0.24
*M. alba* (ethyl acetate) plus cisplatin	160.0	10.0	0.315	0.82
	80.0	10.0	0.486	0.47
	160.0	2.0	0.316	0.81
	80.0	2.0	0.384	0.43
*M. alba* (chloroformic) plus cisplatin	320.0	10.0	0.474	0.55
	160.0	10.0	0.552	0.39
	320.0	2.0	0.460	0.48
	160.0	2.0	0.581	0.23
Isoquercetin plus cisplatin	80.0	10.0	0.516	0.52
	40.0	10.0	0.380	0.48
	80.0	2.0	0.427	0.51
	40.0	2.0	0.324	0.41
Rutin plus cisplatin	80.0	10.0	0.988	0.64
	40.0	10.0	0.904	0.42
	80.0	2.0	0.997	0.61
	40.0	2.0	0.842	0.34

**Table 3:** The single and combination effects of herbal samples with cisplatin on cell cycle phases

**Samples**	**Sub G0 ** **(% cell)**	**G0/G1** **(% cell)**	**S ** **(% cell)**	**G2/M ** **(% cell)**
Negative control (RPMI)	1.2	70.8	16.2	10.1
Solvent	2.8	68.3	14.4	11.5
Cisplatin (2 µg/mL)	16.9	52.2	9.8	18.1
Cisplatin (10 µg/mL)	30.4	45.9	6.3	14.9
*M. alba* (crude)	49.2	39.6	7.2	2.5
*M. alba* (ethyl acetate)	59.2	29.6	4.7	4.2
*M. alba* (chloroformic)	47.9	37.5	5.1	2.7
Isoquercetin	67.8	24.9	3.9	2.1
Rutin	40.1	41.2	9.8	6.3
*M. alba* (crude) + Cisplatin (2 µg/mL)	67.8	23.2	3.2	3.7
*M. alba* (ethyl acetate) + Cisplatin (2 µg/mL)	71.9	20.1	3.8	1.9
*M. alba* (chloroformic) + Cisplatin (2 µg/mL)	50.9	30.4	6.1	6.9
Isoquercetin + Cisplatin (2 µg/mL)	63.1	28.5	4.2	3.1
Rutin + Cisplatin (2 µg/mL)	40.1	40.8	9.1	7.1
*M. alba* (crude) + Cisplatin (10 µg/mL)	70.4	20.6	2.9	3.1
*M. alba* (ethyl acetate) + Cisplatin (10 µg/mL)	86.9	6.8	2.9	1.1
*M. alba* (chloroformic) + Cisplatin (10 µg/mL)	60.9	30.2	5.3	2.3
Isoquercetin + Cisplatin (10 µg/mL)	70.4	18.9	5.7	3.9
Rutin + Cisplatin (10 µg/mL)	52.1	32.7	6.0	5.2

**Table 4:** Antioxidant properties of different *M. alba* extracts

**Samples **	**IC**_50_ ** (µg/mL)** ^1^	**Statistical comparison with the solvent (** ***p *** **< 0.05)**
*M. alba* (crude)	46.044 ± 0.48	*** *p *< 0.001
*M. alba* (ethyl acetate)	32.578 ± 0.51	*** *p *< 0.001
*M. alba* (chloroformic)	81.41 ± 0.53	*** *p *< 0.001
Vitamin C	7.125 ± 0.53	*** *p *< 0.001

^1^ Data shown as mean ± SD
